# The association between dietary omega-3 intake and osteoporosis: a NHANES cross-sectional study

**DOI:** 10.3389/fnut.2024.1467559

**Published:** 2025-01-10

**Authors:** Zhiwen Liu, Shaoming Cai, Yuzhen Chen, Zijing Peng, Huanling Jian, Zhihai Zhang, Hongxing Huang

**Affiliations:** ^1^The Third Clinical Medical College of Guangzhou University of Traditional Chinese Medicine, Guangzhou, China; ^2^The Third Affiliated Hospital, Guangzhou University of Chinese Medicine, Guangzhou, China

**Keywords:** dietary omega-3 intake, bone mineral density, osteoporosis, NHANES, risk factor

## Abstract

**Background:**

Recent research suggests that omega-3 fatty acids may play a role in bone metabolism through their influence on bone mineral density (BMD) and the regulation of bone turnover markers. However, epidemiological evidence linking omega-3 intake to the risk of developing osteoporosis is still emerging and remains inconclusive. This study aims to clarify the role of dietary omega-3 fatty acids in the prevention of osteoporosis.

**Methods:**

We analyzed data from 8,889 participants categorized into normal, osteopenia, and osteoporosis groups based on their BMD scores from the National Health and Nutrition Examination Survey (NHANES). We measured dietary omega-3 intake using two 24-h dietary recall interviews. Dietary omega-3 intake was quantified and divided into quartiles. Multivariate logistic regression and subgroup analysis were used to explore the correlation between dietary omega-3 intake and osteoporosis. The dose–response relationship between the two was analyzed with a restricted cubic spline (RCS).

**Results:**

Higher dietary intake of omega-3 fatty acids was inversely associated with the risk of osteoporosis. Participants in the highest quartile of omega-3 intake had a significantly lower risk (OR = 0.71, 95% CI: 0.53–0.93) compared to those in the lowest quartile, with a consistent trend across all adjusted models (*p* for trend <0.05). Subgroup analyses indicated stronger associations in individuals under 60 years of age, female and non-smokers. In individuals aged under 60, higher omega-3 intake was associated with significantly reduced osteoporosis risk (OR = 0.51, 95%CI: 0.26–0.95), females showed a protective effect of high omega-3 intake against osteoporosis (OR = 0.65, 95% CI: 0.49–0.87). Among non-smokers, higher omega-3 intake was associated with a lower risk of osteoporosis (OR = 0.64, 95% CI: 0.45–0.90), whereas in smokers, the association was not evident (OR = 0.91, 95%CI: 0.55–1.52). No significant associations were found in older participants or smokers. Intake of omega-3 and osteoporosis were linearly related (*p* for nonlinear = 0.366).

**Conclusion:**

This study demonstrates a significant inverse relationship between dietary omega-3 fatty acid intake and osteoporosis risk, suggesting omega-3 s play a crucial role in bone health. However, further longitudinal studies are needed to confirm these findings and refine dietary recommendations for osteoporosis prevention.

## Introduction

Osteoporosis is a significant public health concern that predominantly affects the elderly, leading to increased morbidity and mortality due to fractures, particularly in the hip, spine, and wrist (1, 2). Characterized by reduced bone mass and the deterioration of bone tissue, osteoporosis poses substantial challenges not only for individual health but also in terms of healthcare expenditures (3, 4).

Preventative strategies for osteoporosis have traditionally emphasized the dietary intake of calcium and the maintenance of adequate vitamin D levels, both of which are essential for healthy bone formation (5, 6). Despite these measures, the incidence of osteoporotic fractures has not been fully mitigated, suggesting the involvement of other dietary factors in bone health. In this context, omega-3 fatty acids, known for their anti-inflammatory properties, are garnering interest (7, 8).

Recently, increasing attention has been focused on omega-3 fatty acids. As polyunsaturated fatty acids, omega-3 fatty acids mainly contain alpha-linolenic acid (ALA), eicosapentaenoic acid (EPA) and docosahexenoic acid (DHA), as well as docosapentaenoic acid (DPA). The body obtains omega-3 from fatty deep-sea fish oils, soybean oil, and other foods. These fats are crucial for various physiological functions and have been linked to numerous health benefits, including enhanced cardiovascular health, reduced inflammation, and improved neurological function (9–11). Emerging evidence from epidemiological and experimental studies suggests that omega-3 fatty acids may also positively influence bone metabolism, potentially enhancing bone formation and inhibiting bone resorption through mechanisms involving calcium balance, inflammatory cytokines, and prostaglandins (12, 13).

However, empirical evidence from large-scale population studies examining the link between omega-3 intake and osteoporosis remains limited. Utilizing data from the National Health and Nutrition Examination Survey (NHANES), which provides extensive demographic, dietary, and health-related information, this study investigates the correlation between dietary omega-3 intake and the prevalence of osteoporosis in a diverse US population. The aim is to refine nutritional guidelines and enhance preventive strategies against this debilitating disease.

By exploring the potential connection between omega-3 intake and bone health, this research seeks to expand the scope of dietary recommendations for osteoporosis prevention, underscoring the need for a more comprehensive dietary approach that includes a variety of nutrients. This study hypothesizes that dietary intake of omega-3 fatty acids is closely associated with bone health, particularly through its anti-inflammatory properties and potential effects on bone metabolism, such as regulating osteoblast and osteoclast processes. Although calcium and vitamin D are well-recognized nutrients for bone health, omega-3 fatty acids have gained attention for their unique potential role. Our study focuses on omega-3 to address gaps in existing research on this nutrient’s relationship with bone health.

## Methods

### Study population

The study utilized data from the NHANES, a program designed to assess the health and nutritional status of adults and children in the United States. For this analysis, the study population included participants from the 2005–2010, 2013–2014, 2017–2018 NHANES cycles. Eligible participants were those aged 50 years and older, a demographic typically at increased risk for osteoporosis.

To be included in the study, participants needed to have complete data on dietary omega-3 fatty acid intake from 24-h dietary recall interviews and BMD measurements from dual-energy X-ray absorptiometry (DXA) scans. Both of these components are crucial for determining the association between dietary omega-3 intake and osteoporosis.

Exclusion criteria were missing data on dietary intake, incomplete bone density information, or any medical conditions affecting bone metabolism (e.g., chronic renal failure, hyperparathyroidism) as reported in the NHANES questionnaire. This approach ensured that the analysis was conducted on a cohort with reliable data that could accurately reflect the association between omega-3 intake and osteoporosis risk.

### Assessment of total dietary omega-3 intake

Total dietary omega-3 intake in this study was calculated by summing the intakes of ALA (18:3n-3), DPA (22:5n-3), eicosatetraenoic acid (ETA, 20:4n-3), EPA (20:5n-3), and DHA (22:6n-3). Omega-3 component intake data were obtained from diet-related questionnaires using a 24-h dietary review method and the food frequency questionnaire (FFQ), which follows the automated multiple-pass method (AMPM) and enables the obtained nutritional intake data to differ from the actual intake by within 10%. Food and beverages consumed during the previous 24 h were asked to be recalled during the interview. During the first dietary assessment, professionally trained visitors interviewed the subjects and collected detailed diet information in the previous 24 h. After days 3–10, a telephone call was made to schedule the second visit. Dietary omega-3 intake was calculated as the average intake from the two 24-h dietary review surveys, and only the results of the first survey were used if the subject did not complete both surveys. To correct for potential biases arising from dietary recall errors, we applied regression calibration in our data analysis. Additionally, sensitivity analyses were conducted to assess the robustness of our findings under varying assumptions, thereby enhancing the reliability of the results.

### Diagnosis of osteoporosis

The DXA was used to examine the BMD of lumbar spine and femoral neck. The mean BMD for the scanned anteroposterior length of L1 to L4 was computed and used for lumbar spine BMD reporting. Osteoporosis and osteopenia were defined in the present study using the WHO criteria. Specifically, osteoporosis was defined as a T-score ≤ −2.5 at either the femoral neck or the lumbar spine. Among those without osteoporosis, osteopenia was defined as those with T-scores between −2.5 and −1.0, and normal was defined as T-score ≥ −1. T-scores were calculated as (mean BMD respondent group—mean BMD reference group) / SD reference group. The reference group for calculation of the femoral neck consisted of 20–29 white females from the NHANES III report. As there is no internationally recommended reference group for the lumbar spine, the reference group consisted of 24 normal women from the National Institutes of Health.

Bone loss accelerates with aging, especially in menopausal women; 40% of US White women and 13% of US White men aged > 50 years will experience at least one clinically apparent fragility fracture in their lifetime. Therefore, we set 50 years as the age division for analysis.

### Covariates in NHANES

The covariates were selected from those previously reported factors to influence osteoporosis. Age, race, gender, and body mass index (BMI) are examples of demographic characteristics. The socio-economic covariates include education level, marital status. Covariates on health-related behaviors: smoking and alcohol consumption. Variables related to medical comorbidities: serum calcium, Alanine Aminotransferase (ALT; U/L), Aspartate Aminotransferase (AST; U/L), hypertension, and hyperlipidemia. Missing data in covariates were filled by interpolation.

### Statistical analysis

To summarize the characteristics of the study population, we applied basic descriptive statistics. Continuous variables were described using means and standard deviations, while categorical variables were summarized through frequencies and percentages. Group differences among normally distributed continuous variables, non-normally distributed continuous variables, and categorical variables were evaluated using one-way ANOVA, Kruskal-Wallis tests, and chi-square tests, respectively. The analysis accounted for stratification and weighting to reflect the complex sampling design of NHANES. The relationship between dietary omega-3 intake and osteoporosis risk was analyzed using multivariate logistic regression. Initial adjustments in Model 1 included age, sex, and race. Model 2 extended adjustments to education level and marital status. The comprehensive Model 3 further included variables such as smoking status, BMI, alcohol consumption, serum calcium levels, ALT, AST, hyperlipidemia, and hypertension. Trends in omega-3 intake across quartiles were assessed by treating the median intake of each quartile as a continuous predictor in the models.

Subgroup analyses investigated potential modifying effects of age, sex, and smoking status on the omega-3 and osteoporosis association, with interaction terms tested for statistical significance. Additionally, to mitigate the influence of data extremes, the relationship between omega-3 intake and osteoporosis risk was modeled using restricted cubic splines with knots at the 5th, 35th, 65th, and 95th percentiles, excluding the top and bottom 5% of the data distribution. All statistical procedures were conducted using R statistical software (version 4.3.2), with a significance threshold set at a *p*-value less than 0.05.

## Results

### Participants

**Figure 1:**
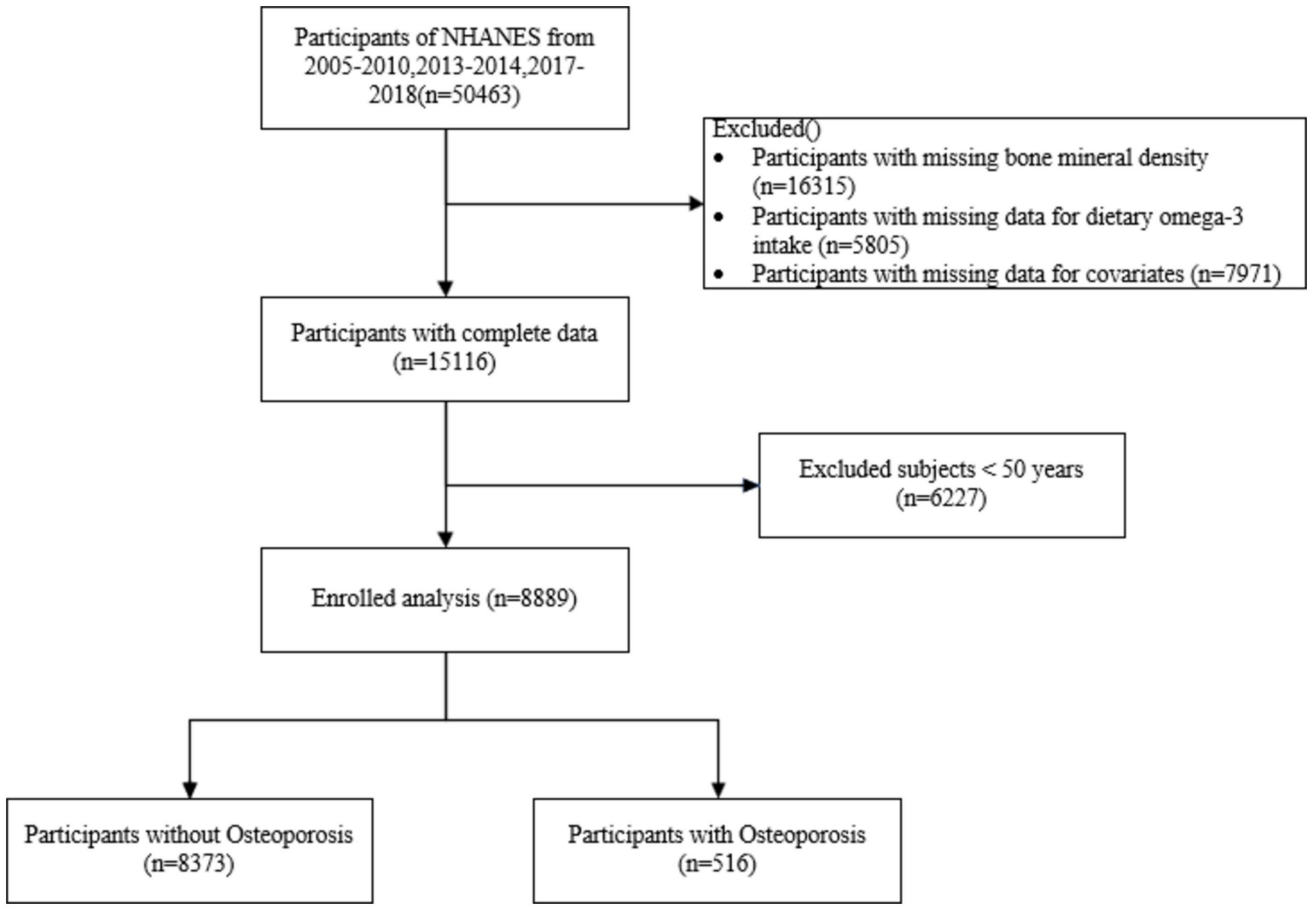
Flowchart of sample selection.

A total of 8,889 study participants were included in this study, of whom 516 (5.80%) were osteoporosis patients and 8,373 (94.2%) were non-osteoporosis patients (Figure 1). Participants aged under 50 years (*n* = 6,227), lack of BMD status (*n* = 16,315), lack of data on dietary omega-3 intake (*n* = 5,805), and lack of individuals with covariates (*n* = 7,971) were excluded.

### Baseline characteristics of participants

Based on the bone health status of participants, Table 1 shows the baseline characteristics of these individuals. This cross-sectional study evaluated 8,889 individuals, stratified into three categories based on their bone density findings: normal bone density (*n* = 4,421), osteopenia (*n* = 3,952), and osteoporosis (*n* = 516). The average age progressively increased across these categories from 62.28 years in the normal group to 71.97 years in the osteoporosis group, demonstrating a clear association between advancing age and deteriorating bone health (*p* < 0.001).The analysis revealed that osteoporosis was predominantly found in females, who made up 80.0% of the osteoporosis category, compared to 20.0% of males, suggesting significant gender disparities in bone degradation risks (*p* < 0.001). Racial distribution showed that Non-Hispanic whites were the most affected by osteoporosis, followed by Hispanic, Non-Hispanic black, and other races, indicating varying susceptibilities among racial groups. Body mass index (BMI) analyses indicated that individuals with osteoporosis were more likely to have a BMI under 25 kg/m^2^ (57.9%), a figure significantly higher than in other bone health categories (*p* < 0.001). Lifestyle behaviors also varied, with osteoporotic individuals less likely to consume alcohol (59.7%) compared to those with normal bone density or osteopenia (*p* < 0.001). A significant decrease in ALT and AST levels was observed in the osteoporosis group (*p* < 0.001). Clinical evaluations showed a decline in liver enzyme levels (ALT and AST) correlating with poorer bone health. However, serum calcium levels remained stable across all groups. Notably, conditions such as hypertension and hyperlipidemia were more prevalent in the osteoporosis group, underscoring their possible influence on bone health deterioration. Regarding dietary habits, omega-3 fatty acid intake was inversely related to bone health status, with the highest intake observed in the normal group (1.86 ± 1.08 g/d) and the lowest in the osteoporosis group (1.66 ± 0.97 g/d), suggesting a potential protective role of omega-3 intake against bone density loss (*p* < 0.001).

**Table 1:** Baseline characteristics of the research population.

Characteristic	Normal(*n* = 4,421)	Osteopenia(*n* = 3,952)	Osteoporosis(*n* = 516)	*p*-value
Age(y)	62.28 ± 8.68	66.31 ± 9.39	71.97 ± 8.82	<0.001
Gender				<0.001
Male	2,900(65.6)	1,669(42.2)	103(20.0)	
Female	1,521(34.4)	2,283(57.8)	413(80.0)	
Race				<0.001
Hispanic	640(14.5)	539(13.6)	38(7.36)	
Non-Hispanic white	1929(43.6)	2,254(57.0)	343(66.5)	
Non-Hispanic black	1,220(27.6)	465(11.8)	37(7.17)	
Other	632(14.3)	694(17.6)	98(19.0)	
Level of education				0.086
Under high school	1,595(36.1)	1,425(36.1)	215(41.7)	
High school or equivalent	1,091(24.7)	1,018(25.8)	119(23.1)	
Above high school	1735(39.2)	1,509(38.2)	182(35.3)	
Marital status				<0.001
Never married	287(6.49)	193(4.88)	24(4.65)	
Married or living with partner	2,925(66.2)	2,418(61.2)	241(46.7)	
Divorced, separated, or widowed	1,209(27.3)	1,341(33.9)	251(48.6)	
BMI (kg/m^2^)				<0.001
<25	664(15.0)	1,334(33.8)	299(57.9)	
25 ~ 29	1,336(30.2)	1,341(33.9)	126(24.4)	
≥30	2,421(54.8)	1,277(32.3)	91(17.6)	
ALT (U/L)	25.78 ± 21.3	22.86 ± 14.08	19.8 ± 13.12	<0.001
AST (U/L)	26.21 ± 15.5	25.09 ± 11.39	24.89 ± 14.83	<0.001
Serum calcium	9.43 ± 0.37	9.44 ± 0.38	9.44 ± 0.42	0.717
Drinking				<0.001
Yes	3,405(77.0)	2,828(71.6)	308(59.7)	
No	1,016(23.0)	1,124(28.4)	208(40.3)	
Smoking				<0.001
Yes	2,336(52.8)	1988(50.3)	218(42.2)	
No	2085(47.2)	1964(49.7)	298(57.8)	
Hypertension				0.006
Yes	2,888(65.3)	2,522(63.8)	365(70.7)	
No	1,533(34.7)	1,430(36.2)	151(29.3)	
Hyperlipidemia				<0.001
Yes	606(13.7)	667(16.9)	93(18.0)	
No	3,815(86.3)	3,285(83.1)	423(82.0)	
T-scores	−0.06 ± 0.77	−1.64 ± 0.40	−2.88 ± 0.35	<0.001
Omega3 intake	1.86 ± 1.08	1.78 ± 1.01	1.66 ± 0.97	<0.001

Mean ± SD for continuous variables: the *p* value was calculated by the weighted linear regression model. (%) for categorical variables: the *p* value was calculated by the weighted chi-square test.

### Relationship between dietary omega-3 intake and bone mineral density

**Figure 2:**
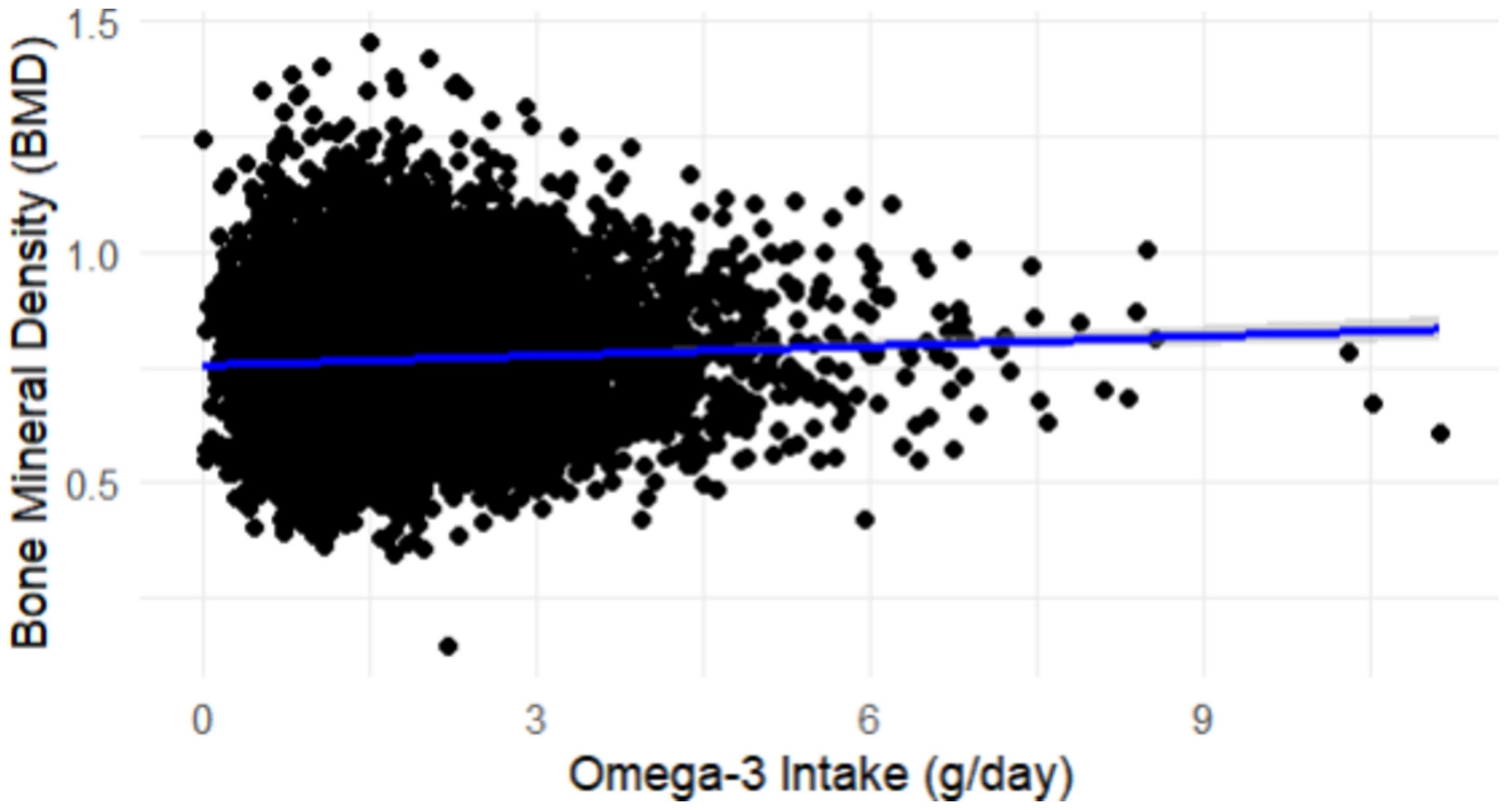
Linear regression analysis showing a modest positive association between Omega-3 intake (g/day) and bone mineral density (BMD), with a 95% confidence interval around the trend line.

To explore the relationship between dietary Omega-3 intake and BMD, a linear regression analysis was performed. The results indicated a modest positive association between Omega-3 intake and BMD, suggesting that higher Omega-3 intake may be linked to slightly improved bone density. As shown in Figure 2, the scatter plot reveals a slight upward trend in BMD with increasing Omega-3 intake, with a regression line indicating the general trend and a 95% confidence interval capturing the variability. While this association was statistically significant (*p* < 0.05), the effect size was relatively small, implying that while Omega-3 intake might contribute to bone health, it is likely one of many factors involved in BMD regulation.

### Relationship between dietary omega-3 intake and osteoporosis

**Figure 3:**
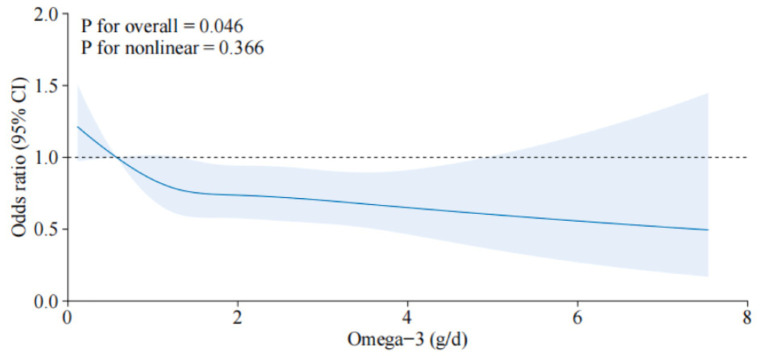
Dose–response relationship between dietary omega-3 intake and osteoporosis. The solid line indicates the estimated risk of osteoporosis, and the dashed line indicates the fitted 95% CI.

The association between dietary omega-3 intake and osteoporosis was rigorously analyzed using data from the NHANES dataset, focusing on a range of omega-3 intake levels divided into quartiles (Table 2). The findings indicate a statistically significant inverse relationship between higher omega-3 intake and the risk of developing osteoporosis, as evidenced by the odds ratios (ORs) across several adjusted models. In the simplest model (Model 1), which adjusted for age, sex, and race, participants in the highest quartile of omega-3 intake (Q4) exhibited a 32% reduction in the odds of osteoporosis compared to those in the lowest quartile (Q1) with an OR of 0.68 (95% CI: 0.52–0.89). This trend was consistent and statistically significant across more comprehensively adjusted models: Model 2, which included adjustments for education level and marital status, showed a similar pattern, with the highest quartile having a 30% reduction in osteoporosis risk (OR = 0.70, 95%CI: 0.54–0.92).Model 3, the most adjusted model incorporating additional factors such as smoking, BMI, alcohol consumption, and various health indicators like serum calcium, liver enzymes (ALT, AST), hyperlipidemia, and hypertension, still showed a protective effect of high omega-3 intake with an OR of 0.71 (95% CI:0.53–0.93).A significant *p*-value for trend (<0.05) across the quartiles in all models suggests a dose–response relationship, where increasing omega-3 intake is associated with progressively lower odds of osteoporosis. Further explorations using restricted cubic splines indicated (Figure 3) a nonlinear relationship between omega-3 intake and osteoporosis risk, though this nonlinear component was not statistically significant (*P* for nonlinear = 0.366). However, the overall effect of omega-3 intake on reducing osteoporosis risk remained significant (*P* for overall = 0.046).

**Table 2:** Odds ratios for associations between dietary omega-3 intake and osteoporosis.

Model	Quartiles of omega-3				*p* for trend
	Q1	Q2	Q3	Q4	
Model 1	1.00	0.70(0.54–0.91)	0.83(0.65–1.07)	0.68(0.52–0.89)	<0.05
Model 2	1.00	0.73(0.57–0.95)	0.84(0.66–1.09)	0.70(0.54–0.92)	<0.05
Model 3	1.00	0.75(0.57–0.98)	0.84(0.65–1.09)	0.71(0.53–0.93)	<0.05

Model 1 was adjusted for age, sex, race. Model 2 was adjusted for age, sex, race, education level, marital status. Model 3 was adjusted for age, race, sex, education level, marital status, smoking, BMI, drinking, serum calcium, ALT, AST hyperlipidemia, hypertension.

### Subgroup analysis of the association between omega-3 intake and osteoporosis

In a comprehensive subgroup analysis using data from NHANES, the relationship between dietary omega-3 intake and osteoporosis was assessed across various demographic and lifestyle factors, including age, sex, race, education, marital status, alcohol intake, smoking status, BMI, hypertension, and hyperlipidemia (Table 3). The analysis, detailed in Model 3, controlled for all variables except the primary stratification factor in each subgroup. Results indicated no significant variations in the association between omega-3 intake and osteoporosis risk across most subgroups (*p* > 0.05 for interaction), suggesting that the protective effects of omega-3 were consistent regardless of sex, race, marital status, and other lifestyle factors. However, age stood out as a significant modifier of the omega-3 and osteoporosis relationship. Younger participants under 60 showed a marked decrease in osteoporosis risk with higher omega-3 intake, whereas this association did not hold for those aged 60 and above, where increased omega-3 intake did not translate to reduced osteoporosis risk (*p* < 0.001 for age interaction). This indicates that the beneficial effects of omega-3 on bone health may be more pronounced in younger populations, highlighting the importance of early dietary intervention.

**Table 3:** Subgroup analysis between dietary omega-3 intake and osteoarthritis risk.

Subgroup	Quartiles of omega-3				*p* for interaction
	Q1	Q2	Q3	Q4	
Age					0.001
<60	1.00	0.50(0.25–0.96)	0.37(0.17–0.74)	0.51(0.26–0.95)	
≥60	1.00	1.49(0.73–3.16)	2.45(1.18–5.42)	1.29(0.65–2.65)	
Sex					0.565
Female	1.00	0.70(0.53–0.92)	0.83(0.67–1.14)	0.65(0.49–0.87)	
Male	1.00	1.23(0.69–2.40)	0.93(0.49–1.73)	1.35(0.73–2.52)	
Races					0.175
Hispanic	1.00	0.45(0.11–1.82)	0.23(0.06–0.80)	0.45(0.12–1.30)	
Non-Hispanic Black	1.00	0.51(0.18–1.33)	1.12(0.48–2.58)	0.80(0.31–1.95)	
Non-Hispanic White	1.00	1.52(0.56–4.58)	0.71(0.29–1.73)	0.69(0.27–1.87)	
Other	1.00	1.59(0.52–5.24)	0.69(0.25–1.89)	0.88(0.30–2.66)	
Education					0.846
Under high school	1.00	1.01(0.56–1.79)	0.91(0.53–1.57)	0.76(0.42–1.37)	
High school or equivalent	1.00	0.96(0.50–1.86)	0.66(0.34–1.28)	0.84(0.43–1.64)	
Above high school	1.00	0.72(0.46–1.12)	0.91(0.61–1.38)	0.72(0.47–1.11)	
Marital status					0.986
Never married	1.00	0.82(0.21–2.73)	1.20(0.37–3.61)	1.37(0.39–4.33)	
Married or living with partner	1.00				
Divorced, separated, or widowed	1.00	0.71(0.50–0.10)	0.75(0.53–1.05)	0.59(0.41–0.86)	
Drinking					0.653
Yes	1.00	1.01(0.61–1.67)	0.78(0.48–1.27)	1.08(0.63–1.87)	
No	1.00	0.71(0.48–1.04)	0.94(0.65–1.35)	0.62(0.39–0.95)	
Smoking					0.089
Yes	1.00	0.96(0.59–1.57)	0.56(0.34–0.91)	0.91(0.55–1.52)	
No	1.00	0.72(0.51–0.99)	0.98(0.72–1.34)	0.64(0.45–0.90)	
BMI					0.676
<30	1.00	0.65(0.50–0.85)	0.77(0.59–0.99)	0.59(0.45–0.77)	
≥30	1.00	1.49(0.74–2.97)	1.00(0.48–2.07)	1.13(0.52–2.39)	
Hypertension					0.688
Yes	1.00	1.16(0.68–1.99)	1.36(0.80–2.31)	1.04(0.61–1.80)	
No	1.00	0.64(0.40–1.00)	0.64(0.40–0.99)	0.60(0.38–0.93)	
Hyperlipidemia					0.608
Yes	1.00	1.16(0.63–2.21)	0.74(0.39–1.39)	0.91(0.45–1.78)	
No	1.00	0.69(0.52–0.90)	0.83(0.64–1.08)	0.63(0.47–0.83)	

Adjusted for age, sex, races, education, marital status alcohol intake, smoking statue, BMI, hypertension, hyperlipidemia, except for subgroup variable.

## Discussion

The association between dietary omega-3 fatty acid intake and reduced osteoporosis risk, as evidenced by this NHANES dataset analysis, aligns with growing evidence that supports the beneficial role of omega-3 s in bone health. This study found a significant inverse correlation, particularly pronounced in individuals under 60 years of age, which echoes findings from several recent epidemiological studies and clinical trials that suggest omega-3 fatty acids can influence bone density and turnover. For instance, Sharma et al. and Smith et al. have reported similar associations (13, 14).

Omega-3 fatty acids, especially EPA and DHA, have been shown to influence bone health by enhancing calcium absorption in the gut—a process crucial for maintaining optimal BMD. This mechanism was elaborately discussed by Waldron, who noted that omega-3 s could alter the lipid composition of cell membranes, thereby affecting calcium channels and enhancing calcium availability for bone tissue (15). Recent studies by Sharma et al. and Poudyal et al. have supported this mechanism, highlighting the role of omega-3 s in improving BMD through enhanced calcium absorption and reduced calcium loss (13, 16).

Furthermore, the anti-inflammatory properties of omega-3 fatty acids are critical in modulating bone turnover. Omega-3 s decrease the production of pro-inflammatory cytokines such as interleukin-6 and tumor necrosis factor-alpha, which are known to stimulate osteoclast activity and bone resorption, as noted by James et al. (17). Moreover, the anabolic effects of omega-3 s on bone may also be mediated through their role in promoting osteoblast activity, enhancing new bone formation. This dual action on bone remodeling is supported by the work of Kruger et al., which demonstrated that dietary omega-3 s significantly influence both bone formation markers and resorption parameters (18). Rahman et al. and Feskanich et al. have provided further evidence of these properties (13, 19).

The results suggest age-related differences in omega-3’s impact on bone health. We hypothesize that these differences may be due to variations in bone turnover rates and calcium absorption with age. The protective effect of omega-3 intake on bone health appears more pronounced in younger individuals, likely due to their higher bone metabolic activity. The pronounced benefits of omega-3 intake observed in younger individuals underpin the potential for age-specific dietary interventions. Studies by Griel et al. and Weiss et al. found similar age-related variations, suggesting that younger adults might more effectively integrate omega-3 fatty acids into cellular structures, including bone (20, 21). In contrast, the metabolic changes associated with aging could diminish the efficacy of omega-3 s in older adults, potentially due to reduced lipid metabolism or alterations in fatty acid incorporation into bone tissues, as reported by MacDonald et al. and Poulsen et al. (22, 23).

The decrease in ALT and AST levels in osteoporosis patients may be related to the anti-inflammatory effects of Omega-3 fatty acids, which are known to influence liver enzyme expression and could indirectly impact bone metabolism. Studies indicate that hypertension and hyperlipidemia are more prevalent among osteoporosis patients, possibly due to the increased inflammatory cytokine levels associated with these metabolic diseases, which may promote bone resorption and reduce bone formation. As an anti-inflammatory nutrient, Omega-3 fatty acids might protect against bone density loss in patients with hypertension and hyperlipidemia.

Recent studies have shown that Omega-3 not only benefits cardiovascular health but may also play a role in osteoporosis prevention through anti-inflammatory and calcium-regulating pathways. This protective effect has been further confirmed in interdisciplinary research, indicating that Omega-3 intake could potentially reduce the risk of osteoporosis. Omega-3 fatty acids may exert protective effects on bone health by inhibiting the expression of pro-inflammatory cytokines such as TNF-*α*, IL-6, and IFN. The overexpression of these cytokines is associated with increased bone resorption, while Omega-3’s anti-inflammatory properties may help mitigate bone loss. Previous studies support this mechanism, suggesting that the anti-inflammatory nature of Omega-3 could positively influence bone metabolism.

The interaction between omega-3 fatty acids and other nutrients critical to bone health, such as vitamin D, magnesium, and vitamin K2, should be considered. Tucker et al. suggested that the synergistic effects of these nutrients with omega-3 fatty acids might enhance their individual impacts on bone density, proposing a holistic approach to dietary recommendations for osteoporosis prevention (24). Recent studies by Smith et al. and Appleton et al. have shown that dietary supplementation combining omega-3 s with vitamin D and calcium can provide significant improvements in BMD compared to supplementation with either nutrient alone (14, 25).Given the increasing prevalence of osteoporosis, particularly among postmenopausal women and the elderly, embedding omega-3 fatty acids in dietary guidelines could provide a viable strategy to reduce the incidence of osteoporosis. Public health campaigns should focus on promoting omega-3-rich foods and possibly advocating for supplementation, especially in populations less likely to consume adequate amounts through diet alone.

Longitudinal studies and randomized controlled trials are essential to confirm the causal relationships suggested by cross-sectional data. Recent trials by Rahman et al. and Panagiotakos et al. have indicated promising results, but more extensive and longer-term studies are needed to establish definitive guidelines. Moreover, exploring the genetic factors that affect individual metabolism of omega-3 fatty acids might provide insights into personalized dietary recommendations, which could enhance the precision of nutritional interventions for osteoporosis prevention, as proposed by Darling et al. and Panagiotakos et al. (26, 27).

## Limitations

This study has several limitations. First, the reliance on self-reported dietary data may introduce recall bias, potentially affecting the accuracy of Omega-3 intake measurements. Second, the cross-sectional design limits the ability to establish causality, as it captures associations at only one point in time. Third, the findings may not be generalizable to all populations, as the sample was drawn from specific demographic groups. Despite these limitations, the study provides insights into the potential role of Omega-3 in supporting bone health.

## Conclusion

This study highlights the significant inverse relationship between dietary omega-3 fatty acid intake and osteoporosis risk. The findings suggest that omega-3 fatty acids may play a critical role in bone health, supporting the need for dietary recommendations that encourage omega-3 consumption as a preventive measure against osteoporosis, such as fatty fish, flaxseeds, and walnuts, especially for middle-aged and younger individuals, as their higher bone metabolic activity may allow them to derive greater benefits. This dietary recommendation not only has potential benefits for osteoporosis prevention but also carries public health implications.

## Data Availability

The datasets presented in this study can be found in online repositories. The names of the repository/repositories and accession number(s) can be found in the article/supplementary material.
